# De Novo Assembly and Characterization of the Transcriptome of Grasshopper *Shirakiacris shirakii*

**DOI:** 10.3390/ijms17071110

**Published:** 2016-07-22

**Authors:** Zhongying Qiu, Fei Liu, Huimeng Lu, Hao Yuan, Qin Zhang, Yuan Huang

**Affiliations:** 1 College of Life Sciences, Shaanxi Normal University, Xi’an 710062, China; qiuzhongying11@126.com (Z.Q.); liufei@snsy.edu.cn (F.L.); yuanhao@snnu.edu.cn (H.Y.); shengkezhangqin@126.com (Q.Z.); 2 College of Life Sciences and Food Engineering, Shaanxi Xueqian Normal University, Xi’an 710061, China; 3 Key Laboratory for Space Bioscience & Biotechnology, School of Life Sciences, Northwestern Polytechnical University, Xi’an 710072, China; luhuimeng@nwpu.edu.cn

**Keywords:** *Shirakiacris shirakii*, transcriptome, insecticide resistance, RNAi, differentially-expressed unigenes (DEUs)

## Abstract

Background: The grasshopper *Shirakiacris shirakii* is an important agricultural pest and feeds mainly on gramineous plants, thereby causing economic damage to a wide range of crops. However, genomic information on this species is extremely limited thus far, and transcriptome data relevant to insecticide resistance and pest control are also not available. Methods: The transcriptome of *S. shirakii* was sequenced using the Illumina HiSeq platform, and we de novo assembled the transcriptome. Results: Its sequencing produced a total of 105,408,878 clean reads, and the de novo assembly revealed 74,657 unigenes with an average length of 680 bp and N50 of 1057 bp. A total of 28,173 unigenes were annotated for the NCBI non-redundant protein sequences (Nr), NCBI non-redundant nucleotide sequences (Nt), a manually-annotated and reviewed protein sequence database (Swiss-Prot), Gene Ontology (GO) and Kyoto Encyclopedia of Genes and Genomes (KEGG) databases. Based on the Nr annotation results, we manually identified 79 unigenes encoding cytochrome P450 monooxygenases (P450s), 36 unigenes encoding carboxylesterases (CarEs) and 36 unigenes encoding glutathione *S*-transferases (GSTs) in *S. shirakii.* Core RNAi components relevant to miroRNA, siRNA and piRNA pathways, including Pasha, Loquacious, Argonaute-1, Argonaute-2, Argonaute-3, Zucchini, Aubergine, enhanced RNAi-1 and Piwi, were expressed in *S. shirakii*. We also identified five unigenes that were homologous to the *Sid-1* gene. In addition, the analysis of differential gene expressions revealed that a total of 19,764 unigenes were up-regulated and 4185 unigenes were down-regulated in larvae. In total, we predicted 7504 simple sequence repeats (SSRs) from 74,657 unigenes. Conclusions: The comprehensive de novo transcriptomic data of *S. shirakii* will offer a series of valuable molecular resources for better studying insecticide resistance, RNAi and molecular marker discovery in the transcriptome.

## 1. Introduction

*Shirakiacris shirakii*, which belongs to Orthoptera, Caelifera, Acridoidea and Catantopidae, is widely distributed and is an important agricultural pest. *S. shirakii* primarily feeds on the sap of gramineous plants, bringing about considerable damage to crops, particularly Gramineae, Leguminosae, and Compositae. Control strategies for this pest traditionally rely on broad spectrum insecticides and are often the cause of insecticide resistance. Due to a lack of genetic information, previous studies on *S. shirakii* mostly focused on its morphology and the basic biological and genetic characteristics, such as chromosome localization research based on fluorescence in situ hybridization (FISH) [1] and allozyme analysis [2]. Moreover, only small numbers of putative gene sequences for *S. shirakii* are available in GenBank, including 14 nucleotide sequences and 35 protein sequences (June 2016).

A large number of commercial insecticides are used worldwide [3]. However, frequent insecticide applications often induce tolerance through mutation by reducing the binding of the insecticide to its targets, the sequestration of insecticides and a reduced penetration of the insecticide. Insecticide resistance-related proteins include cytochrome P450 monooxygenases (P450s), carboxylesterases (CarEs) [4] and glutathione *S*-transferases (GSTs) [5]. RNAi is a widely-used and powerful biological tool for knocking down and analyzing the function of genes in many eukaryotic systems and is especially important in non-model organisms [6]. RNAi has been proposed to be a potential tool for crop protection against insect pests in agriculture [7,8]. This technique has been described in various insect orders, including Orthoptera [9,10,11], and *T. castaneum* was reported as a model organism for RNAi [12].

Recently, transcriptome sequencing has been widely used, especially in non-model organisms when genome information is not available [13]. By de novo transcriptome sequencing of *S. shirakii*, we sought to gain a preliminary understanding of mRNAs that might be associated with RNAi pathways, to provide useful information for grasshopper pest control and to obtain large amounts of sequence data that could be used to analyze the molecular mechanisms of insecticide resistance.

The standard experimental methods for simple sequence repeat (SSR) markers’ identification are time consuming and expensive. Transcriptome sequencing provides a good method to obtain SSRs. SSRs from numerous species have been obtained by transcriptome sequencing, given its high throughput [14,15]. In the present study, we identified the SSRs of *S. shirakii.*

Here, we report a de novo transcriptome of the grasshopper, *S. shirakii*, confirmed the suitability of the de novo assembly and compared the gene expression profiles between larvae and adults. These data enriched the transcriptome resources of Orthopteran insects and offered a valuable molecular resource for a better understanding of both *S. shirakii* insecticide resistance and RNAi machinery, as well as for facilitating molecular marker discovery.

## 2. Results

### 2.1. Transcriptome Assembly

After filtering low quality reads, a total of 105,408,878 clean reads was retained (Table 1) with 97.42% and 97.45% of the Q20 value in the two transcriptomes. A total of 135,320 contigs from the adult female sample with a mean contig size of 290 bp and N50 of 428 bp was produced by the Trinity software, as well as 187,282 contigs of the larval female sample with a mean contig size of 292 bp and N50 of 445 bp (Table 1). Then, the contigs were assembled into 93,948 unigenes for the larvae sample and 69,189 unigenes for the adult sample, respectively. The final assembly, which combined two transcriptomes, resulted in 74,657 unigenes, with an average length of 680 bp and N50 of 1057 bp (Table 1). These unigenes consist of 16,298 clusters and 58,359 singletons.

To demonstrate the accuracy of the unigenes, the submitted 14 nucleotide sequences of *S. shirakii* from GenBank were searched against transcriptome unigenes with BLASTN with an *E*-value of 10^−5^. The results showed that all of the sequences of *S. shirakii* in the NCBI database were represented in the transcriptome (Table S1). Analysis of the assembled unigenes using the BUSCO program identified 1388 of the 2675 core proteins (63%) as complete and a fragmented score of 14% (Table S2).

### 2.2. Functional Annotation

BLASTX and BLASTN similarity searches were conducted between the assembly of unigenes and the following public databases: the NCBI non-redundant protein sequences (Nr), NCBI non-redundant nucleotide sequences (Nt), a manually annotated and reviewed protein sequence database (Swiss-Prot), Gene Ontology (GO) and Kyoto Encyclopedia of Genes and Genomes (KEGG) databases, respectively. Table 2 presents the number of unigenes annotated to these six public databases.

### 2.3. Nr Annotation

**Figure 1:**
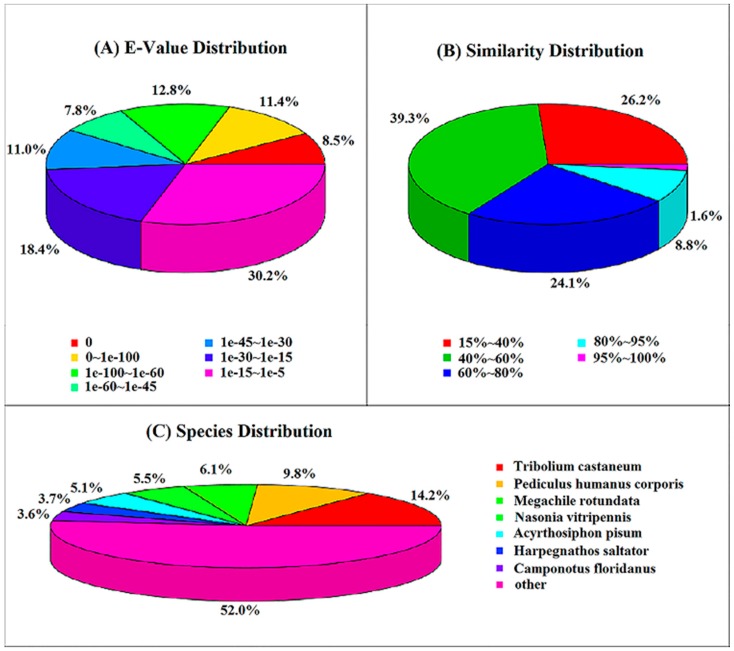
Homology analysis of unigenes for *S. shirakii*. (**A**) *E*-value distribution of the top BLASTX hits against the Nr database for *S. shirakii* unigenes with an *E*-value cutoff of 10^−5^; (**B**) Similarity distribution of the top BLAST hits for each sequence; (**C**) Species distribution of the top *S. shirakii* sequence BLASTX hits. BLAST analysis against the non-redundant database was performed with an *E*-value cutoff of 10^−5^.

In total, 25,652 (34.36% of all distinct sequences) unigenes had Nr hits with BLASTX similarity search with the cut-off *E*-value of 10^−5^. Nr annotation and the *E*-value distribution indicated that 40.4% of the mapped sequences showed strong homology (*E*-value ≤ 10^−45^), whereas 59.6% of the homolog sequences ranged from 1.0 × 10^−5^–1.0 × 10^−45^ (Figure 1A). For the similarity distribution, 34.5% hits had a similarity over 60%, only 10.4% of sequences having a similarity higher than 80% (Figure 1B). For the species distribution, sequences of *T. castaneum* (Coleoptera, Tenebrionidae) were most commonly the top hit for sequences of *S. shirakii* (3649 unigenes, 14.2%). The following were *Pediculus humanus corporis* (9.8%), *Megachile rotundata* (6.1%), *Nasonia vitripennis* (5.5%), *Acyrthosiphon pisum* (5.1%), *Harpegnathos saltator* (3.7%) and *Camponotus floridanus* (3.6%) (Figure 1C).

### 2.4. GO, COG Classification, Swiss-Prot and KEGG Pathway

In total, 28,173 of the 74,657 unigenes were annotated in the databases. Among these unigenes, a total of 6044 unigenes were annotated by the GO, COG classification, Swiss-Prot and KEGG pathway databases (Table 2, Figure S1).

**Figure 2:**
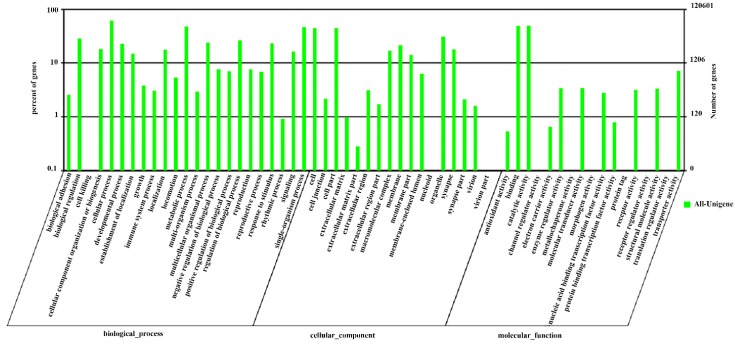
Gene Ontology. Classification of *S. shirakii* unigenes based on Gene Ontology (GO).

Among the 25,652 hits in the Nr database, 12,061 unigenes (47%) were assigned to one or more GO terms and 57 subcategories using Blast2GO based on BLASTX similarity searches [16]. GO includes three main functional categories: biological process, molecular function and cellular component. In addition, the terms “cellular process”, “catalytic activity”, “metabolic process”, “single-organism process” and “binding” are dominant; However, only a few genes from the “cell killing”, “virion”, “virion part” and “protein tag” terms were annotated (Figure 2).

**Figure 3:**
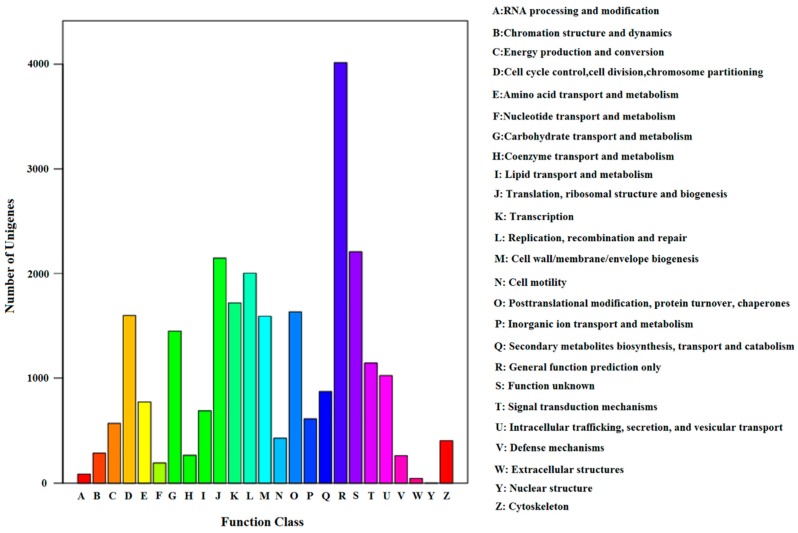
Clusters of orthologous group (COG) function classification of the *S. shirakii* unigenes.

To gain a more detailed annotation of the transcriptome, we assigned COG classification terms. In total, 9558 *S. shirakii* unigenes had a COG classification (Figure 3). The top five groups were as follows: “a general function prediction only” (4014, 42.0%), “function unknown” (2208, 23.1%), “translation, ribosomal structure and biogenesis” (2149, 22.5%), “replication, recombination and repair” (2003, 20.9%) and “transcription” (1718, 17.9%). Among these, 23.1% of unigenes were classified as “function unknown” groups, which indicated that there could be many unique or novel genes in the *S. shirakii* transcriptome. “Extracellular structures”, “nuclear structure” and “RNA processing and modification” consisted of 44, 4 and 88 unigenes, respectively, representing the smallest COG groups (Figure 3).

A total of 18,053 unigenes was mapped to the metabolism, genetic information processing, environmental information processing, cellular processes and organismal systems pathways in the KEGG pathway database (Table 3). Among these pathways, metabolic pathways (2520 unigenes), signal transduction (1603 unigenes) and digestive system (1399 unigenes) contained the largest number of unigenes.

### 2.5. Differentially-Expressed Unigenes’ Analysis

**Figure 4:**
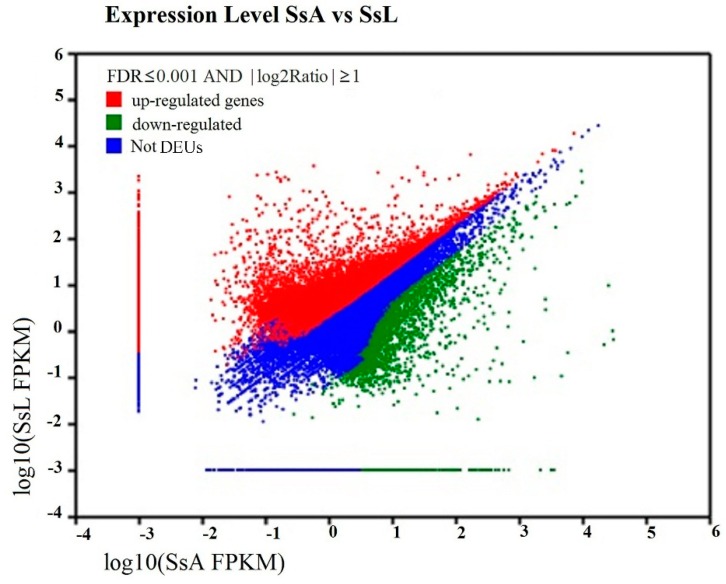
Comparison of unigene expression between adults and larvae of *Shirakiacris shirakii.* The differentially-expressed unigenes (DEUs) are shown in red and green, while blue indicates unigenes that were not differentially-expressed between the adults and larvae of *S. shirakii.* SsA represents the adults of *S. shirakii*; *Ss*L represents the larvae of *S. shirakii*.

The results of the differentially-expressed unigenes (DEUs)’ comparison between the adults and larvae showed that a total of 19,764 unigenes were annotated to be up-regulated, and 4185 unigenes were down-regulated in the larvae (Figure 4). The top fifty up- and down-regulated expressed genes are listed in Table S3. Among the fifty top up-regulated genes, 34 DEUs have predicted functions, i.e., the putative *Apolipoprotein D* precursor (*Pediculus humanus corporis*), *CPG12* (*Papilio xuthus*), *UDP-N-acetylglucosamine transferase subunit ALG13 homolog* (*Equus caballus*) and *vitellogenin* (*Vg*) (*Athalia rosae*). A total of 47 unigenes of the top fifty up- and down-regulated unigenes was blasted against the Nr database without annotation with an *E*-value of 10^−5^.

All of the DEUs were aligned to the GO database. In total, 4447 DEUs were assigned to GO terms by Blast2GO annotation (Table S4). Among these, a total of 3256 DEUs was mapped to the “biological process” category. A total of 3258 DEUs was assigned to the “molecular function” category, and 2464 DEUs were assigned to the “cell component” category. Cellular process (2633), metabolic process (2181 unigenes) and single-organism process (1950 unigenes) represented a high percentage of the biological process categories. Regarding the cellular component category, cell (2057 unigenes), cell part (2057 unigenes) and organelle (1441 unigenes) were highly represented. Catalytic activity (2255 unigenes) and binding (2139 unigenes) differed mostly in the molecular function category.

### 2.6. Insecticide Resistance

**Figure 5:**
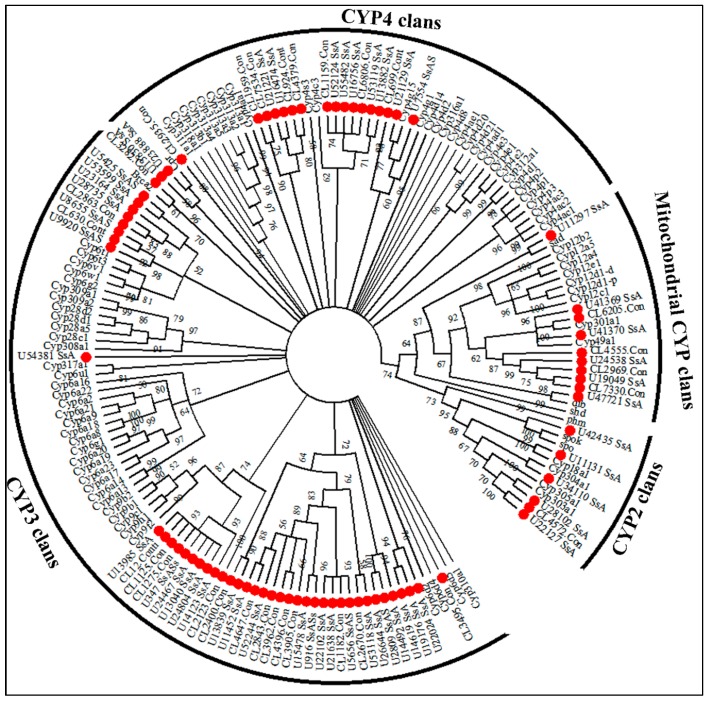
The phylogenetic analysis of sequences encoding P450s from *S. shirakii* and *D. melanogaster*. Branch numbers represent bootstrap values (1000 replicates). The 79 *S. shirakii* unigenes encoding P450s are marked with filled red circles. The sequences used to reconstruct the maximum likelihood (ML) tree are available as S1 Data.

**Figure 6:**
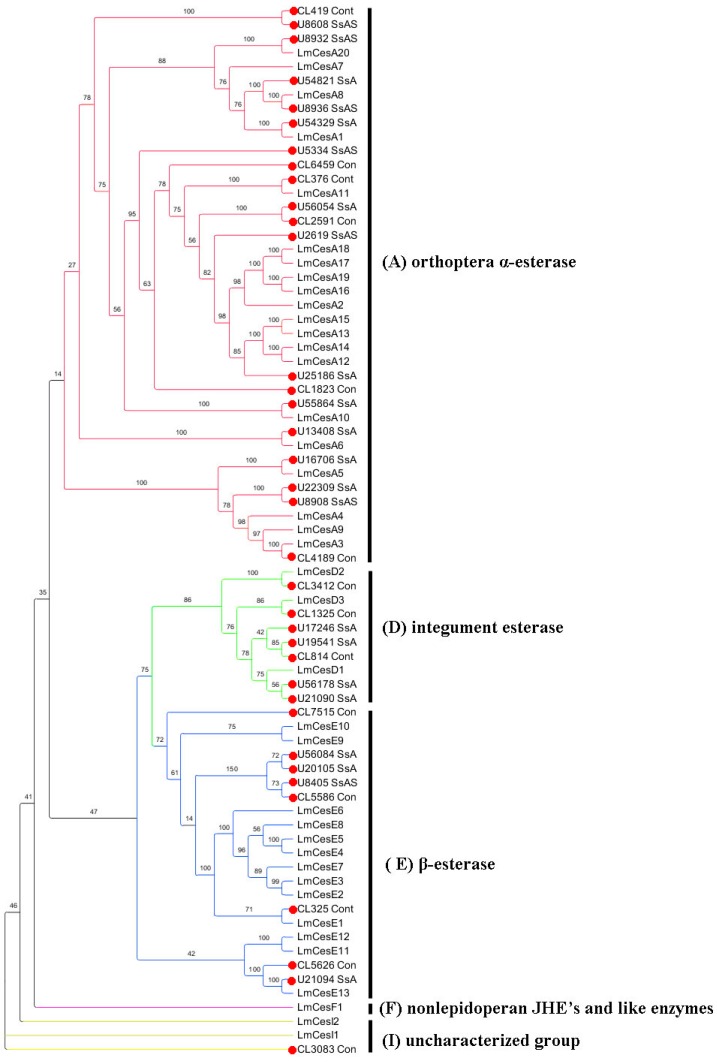
The phylogenetic analysis of sequences encoding CarEs from *S. shirakii* and *L. migratoria*. Branch numbers represent bootstrap values (1000 replicates). The 36 *S. shirakii* unigenes encoding CarEs are marked with filled red circles. The sequences used to reconstruct the maximum likelihood (ML) tree are available as S2 Data.

Based on the Nr annotation results, we manually selected a number of unigenes that were homologous to genes related to insecticide resistance. A total of 198, 46 and 64 unigenes corresponding to P450-, GST- and CarE-related unigenes were identified separately from the Nr annotation in the *S. shirakii* transcriptome. After removing redundant and short sequences (sequences in CarEs and P450s with lengths <500 bp and sequences in GSTs with lengths <200 bp were considered as short sequences), we obtained 79, 36 and 36 unigenes that putatively encode P450s, CarEs and GSTs (Tables S5–S7), respectively. Figure 5 shows the phylogenetic tree constructed based on amino acid sequences deduced from 79 unigenes encoding P450s from *S. shirakii* and 90 genes encoding P450s from *Drosophila melanogaster*. According to the *D. melanogaster* P450s superfamily, all CYP genes can be divided into four clans: CYP2, CYP3, CYP4 and the mitochondrial CYP clans (M) [17]. The phylogenetic tree analysis revealed that the majority of *S. shirakii* P450s-encoding genes belonged to clan 3 (44) and clan 4 (19) compared to clan 2 (six) and clan M (10) (Figure 5 and Table S5). Figure 6 shows the phylogenetic tree constructed based on amino acid sequences from 36 putative CarEs-encoding genes from *S. shirakii* and 39 CarEs-encoding genes from *L. migratoria*. Our 36 *S. shirakii* CarEs-encoding genes were distributed into five clades, including clade A with *α-esterase* (20 unigenes), clade D with *integument esterase* (seven unigenes), clade E with *β-esterases* (eight unigenes), clade F with nonlepidopteran JHE’s and like enzymes-related genes (zero unigene) and clade I with uncharacterized *esterases* (one unigene) (Figure 6 and Table S6). The 36 putative unigenes encoding GSTs were assigned to eight cytosolic classes (*Epsilon* (four unigenes), *Delta* (13 unigenes), *Omega* (three unigenes), *Theta* (two unigenes), *Sigma* (eight unigenes), *Zeta* (one unigene), *GSTA* (one unigene)) and microsomal GST encoding genes (four unigenes) (Figure S2 and Table S7).

### 2.7. Core RNAi Components

Two cofactors, Pasha and Loquacious (Loqs), were identified in *S. shirakii* (Table 4, Table S8, and S4). These two proteins are required to interact with the RNaseIII Drosha, Dicer-1 and Dicer-2. In *Drosophila*, R2D2 acts as a bridge between the initiation and effector steps of the RNAi pathway by facilitating siRNA passage from Dicer to RNA-induced silencing complex (RISC). However, the *R2D2* gene was not detected in *S. shirakii*.

Two orthologs of *Argonaute* (*Ago*) genes, *Argonaute*-1 (*Ago-1*) and *Argonaute*-2 (*Ago-2*), were identified. These genes are involved in miRNA and siRNA pathways, respectively, and exhibited high homology to the counterparts of *L. migratoria* (AGO85968 and AGO85972). Argonaute-3 (Ago-3), Zucchini, Aubergine (Aub) and Piwi are proteins involved in piRNA [18]. The *Piwi* gene is present in *L. migratoria* and *S. shirakii* (Table 4 and Table S8). The *Zucchini* and *Ago-3* genes were also identified in *S. shirakii*. In addition, we also identified the *Sid-1* gene by a TBLASTN homology search using the *L. migratoria* SID-1 (AFQ00936) protein sequence as a query sequence. In total, we identified five unigenes that were homologous to the *Sid-1* gene (Table 4 and S4). These five unigenes are blasted to different parts of the *L. migratoria Sid-1* gene.

### 2.8. Simple Sequence Repeats (SSRs)

**Figure 7:**
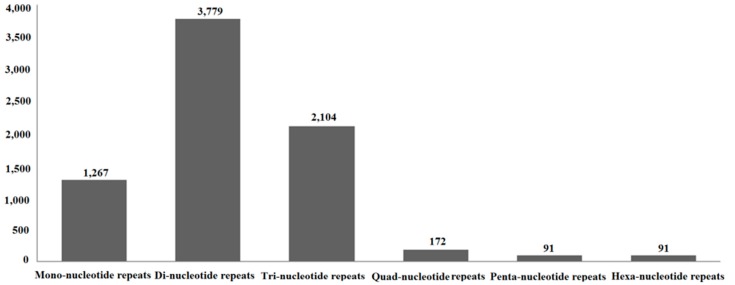
Statistics of simple sequence repeat (SSR) nucleotide classes found in the transcriptome of *S. shirakii.*

In total, 6655 sequences containing 7504 SSRs were predicted from 74,657 unigenes. The SSR frequency was 8.91% (6655/74,657), and the distribution rate was 10.05% (7504/74,567) in the *S. shirakii* transcriptome. Among them, 730 sequences contained more than one SSR, and dinucleotide repeats (50.36%) represented the most abundant microsatellite repeat units, followed by trinucleotide (28.04%), mononucleotide (16.88%), quadranucleotide (2.29%), pentanucleotide (1.21%) and hexanucleotide (1.21%) repeats (Figure 7 and Table S9).

### 2.9. Quantitative Real-Time PCR Results

**Figure 8:**
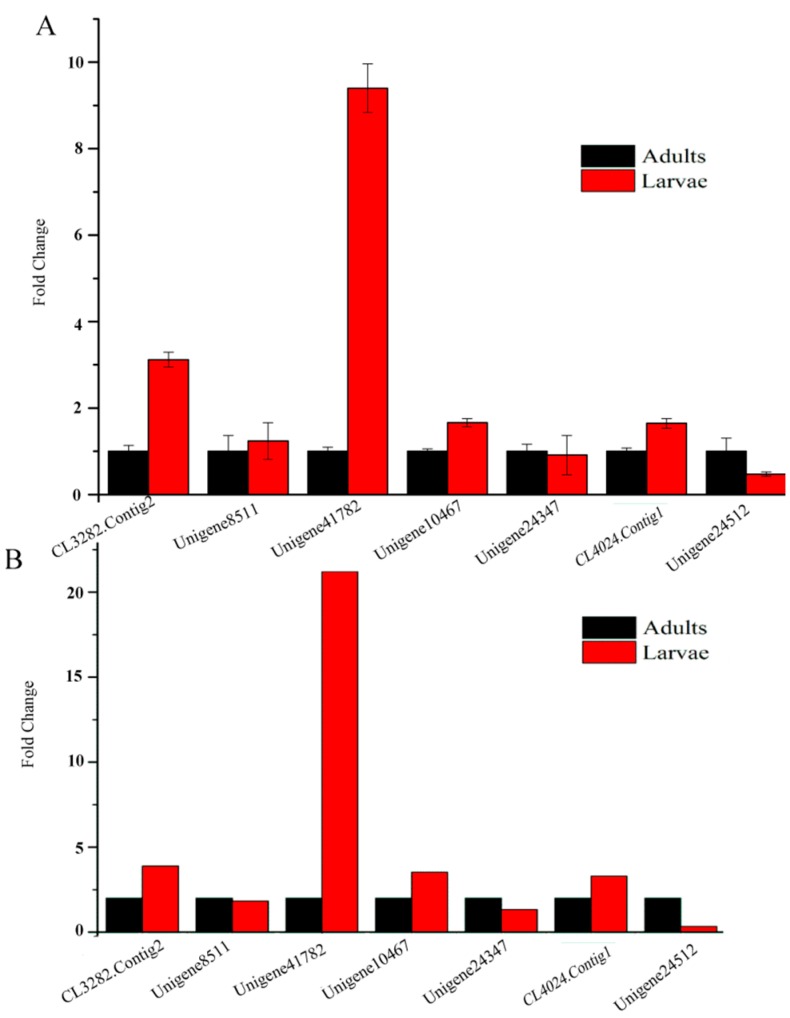

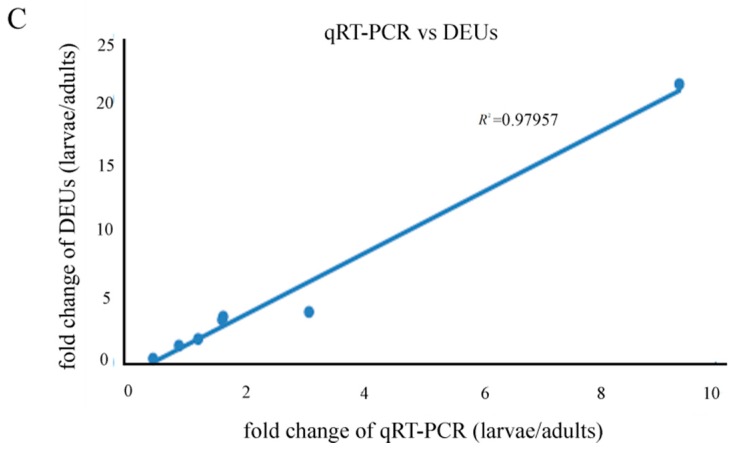
Differential expression of DEUs in *S. shirakii*. (**A**) Gene expression data obtained by qRT-PCR analysis. Expression ratios of seven genes in SsL compared to SsA; (**B**) The fold changes of the genes were calculated as the SsL/SsA comparison and are shown on the y-axis. SsA represents the adults of *S. shirakii; Ss*L represents the larvae of *S. shirakii*; (**C**) Correlation between the expression fold change level of DEUs between adults and larvae.

To validate our transcriptome data, we randomly chose seven unigenes, designed primers and used the *gapdh* gene as the control to measure their expression in the same RNA sample of adults and larvae by qRT-PCR. All seven unigenes’ results showed uniformly consistent results in RT-PCR with RNA-Seq (Figure 8), which indicates that transcriptome sequencing was reliable and that we could make reasonable deductions from the functional enrichment analysis of the DEUs.

## 3. Discussion

The *S. shirakii* transcriptome generated 74,657 unigenes, and their annotation information from the Nr, Nt, COG, GO, KEGG and Swiss-Prot databases provided valuable resources for molecular studies of *S. shirakii*. However, a large number of unigenes (62.26%) unmatched to Nr, Nt, GO, COG, KEGG and Swiss-Prot databases are short sequences or potentially novel genes [19,20,21]. Many differentially-expressed genes achieved by comparative transcriptomic analyses between the samples greatly enriched the current knowledge of *S. shirakii* gene expression profiles. Most of the differentially-expressed genes were up-regulated at the larvae stage when compared to the adult stage. In contrast, most of these genes were down-regulated in adults. We analyzed the top fifty up-and down-regulated expressed unigenes between samples (larvae vs. adults) (Table S3). Among the top fifty up- and down-regulated DEUs, 15 DEUs had exact annotations. Most of these DEUs were related to innate immune system, pathogen invasion and transcription. Vg is the precursor of vitellin (Vn), which plays a vital role in oocytes and embryo development in oviparous animals, such as insects [22]. In the present study, the *Vg* gene (log2 adult/larvae = 15.5346) was up-regulated in adults compared to larval samples. It was reasonable that adult samples were collected in August, and the females had their eggs. Ova require nutrition, and Vg could support the nutritional needs. However, of the top 50 up- and down-regulated DEUs, 47 DEUs were found to be non-homologues in the NCBI Nr database. Most defined genes are annotated to a hypothetical or uncharacterized protein, likely due to the lack of detailed molecular information for *S. shirakii*. This phenomenon is consistent with the whitefly (*Bemisia tabaci*) transcriptome, and it is expected that Orthopteran genes are expressed during development that have no homologs in other species [23].

P450s, CarEs and GSTs were reported to have a role in developing insecticide resistance with respect to metabolic and detoxification processes. In insects, more than 1000 P450s genes have been identified, and the numbers of genes among insect species vary considerably (48 genes in *Apis mellifera*, 94 genes in *L. migratoria* and 164 genes in *Aedes aegypti*). P450s play many functional roles in insect growth, development and the inactivation and metabolism of xenobiotic compounds, such as pesticides. The mitochondrial CYP clan in insects is involved in xenobiotic metabolism [24]. The CYP2 clan in insects includes P450s with essential physiological functions, e.g., ecdysone metabolism and juvenile hormone biosynthesis [25]. Considerable evidence links members of the CYP3 clan in insects to xenobiotic metabolism and insecticide resistance. In contrast, some CYP4s appear to be inducible metabolizers of xenobiotics, and others have been linked to odorant or pheromone metabolism [24]. CarEs belong to superfamily enzymes that can hydrolyze the carboxyl ester bond and the phosphodiester bond, thus metabolizing various ester bond-containing hormones, pheromones, drugs and insecticides. Based on the phylogenetic analysis in *S. shirakii*, *S. shirakii* unigenes encoding CarEs were divided into five clades, and most of unigenes encoding CarEs were identified to be clade A of the dietary detoxification group (20 *α-esterase* genes) and clade E (eight *β-esterase* genes) of the hormone/semiochemical-processing group. This phenomenon was consistent with the CarEs superfamily in *L. migratoria*. This finding might be why, as described in *L. migratoria*, abundant detoxification genes can be used for the digestion of many different plant secondary metabolites and for developing insecticide resistance [26]. *LmCesA20* silencing increased the nymphal mortalities in *L. migratoria*, which plays an important role in the detoxification of malathion in the locust [26]. In our research, *Unigene8932_SsASsL* and *LmCesA20* are sister groups in Figure 6. The identity of these two sequences is 72% with an *E*-value of 3 × 10^−173^. We inferred that *Unigene 8932_SsASsL* might play an important role in the detoxification of malathion and might be a potential target for pest control. In total, 32 unigenes encoding cytosolic GSTs) and four unigenes encoding microsomal GSTs were identified from the transcriptome of *S. shirakii* according to *L. migratoria*’s GSTs-encoding genes [5]. The estimated numbers of unigenes encoding P450s, CarEs and GSTs genes may be lower than the actual numbers in the locust genome, given the limitations of transcriptome technology. These results are expected to help researchers reveal the characteristics of diverse P450s, GSTs and CarEs.

RNAi is a widely-used gene-silencing tool in insects for embryogenesis, pattern formation, reproduction, biosynthesis, pest control and behavior [27]. However, there are many limitations to its application [7]. An RNAi-based strategy in pest control has been used in insects, including species of Orthoptera [28]. The identified genes, namely P450s-, CarEs- and GSTs-encoding genes, may facilitate the development of novel control strategies for *S. shirakii* and other Orthoptera insects. CYP4, 6, 9 and 12 family members have frequently been linked to insecticide metabolism and resistance [29,30,31]. The CYP6 family is unique to the class Insecta, and its biochemical function is associated with the metabolism of xenobiotics. *CYP6FF1*, *CYP6FD2* and *CYP6FE1* silencing resulted in significant mortality of *L. migratoria* nymphs to deltamethrin and carbaryl [31]. *CYP6B* P450s may contribute to deltamethrin metabolism in the cotton bollworm [32]. Silencing *CYP6B6* expression reduced the resistance to pyrethroids of cotton bollworm and significantly increased the bollworm larval mortality rate significantly [33]. Tang et al. reported that silencing of *CYP6B7* alone or *CYP6B7* together with *CPR* and/or *Cyt-b_5_* increased the susceptibility of *H. armigera* to fenvalerate [34]. RNAi of *LmCesA1* and *LmCesA2* increased insect mortalities by 20.9% and 14.5%, respectively, when chlorpyrifos was applied. These results suggested that these genes might not play a significant role in the detoxification of carbaryl and deltamethrin, but are most likely to be involved in the detoxification of chlorpyrifos in *L. migratoria* [4]. The nymph mortalities increased from 34.3% to 65.2% and 54.2%, respectively, after *LmCarE9* and *LmCarE25* silencing increased the nymph mortalities in the locust [35]. Silencing of an aphid carboxylesterase gene using plant-mediated RNAi impairs *Sitobion avenae* tolerance of Phoxim [36]. Thus, RNAi is a reliable molecular tool that offers great promises in meeting the challenges imposed by crop insects with the careful selection of key enzymes/proteins [37]. Moreover, the locust is a good model to study the regulatory mechanisms of RNAi in insects, particularly in *S. gregaria* and *L. migratoria*, displaying a highly robust and sensitive systemic RNAi (sysRNAi) response with tissue-dependent reduction of RNAi potency in reproductive organs [9,10]. The core RNAi components related to siRNA, miRNA and piRNA of the RNAi pathway, as identified in *S. shirakii*, provide a molecular basis for other Orthopteran species (Table 4).

SID-1 has been reported to be the best characterized protein involved in sysRNAi in *C. elegans* [38], but not in *D. melanogaster*, which lacks an endogenous SID-1 ortholog and does not exhibit a robust sysRNAi response. The presence of a *Sid-1-like* gene is hypothesized to be the determinant of whether sysRNAi occurs in an organism. The number of *Sid-1* gene copies varies among insects. Hemipterans, Hymenopterans, Orthopterans and Phthirapterans only have one *Sid-1* gene [12,39], Three homologs of *C. elegans* *Sid-1* were identified in *T. castaneum*, and *B. mori* (Lepidoptera) has three *Sid-1-like* genes [12]. In the *L. migratoria*, SID-1 is not necessary for sysRNAi [9]; however, this species exhibits a robust sysRNAi response. The *Sid-1-like* gene in *T. castaneum* shared more identity with the *C. elegans* gene, *tag-130*. The *tag-130* gene in *C. elegans* was not required for sysRNAi [12]. This suggested that the *Sid-1* gene was not functional for sysRNAi and had a function similar to the *tag-130* in other pathways in *S. shirakii*. Insect sysRNAi had alternative mechanisms.

In addition, there were no orthologous genes for RNA-dependent RNA polymerase (RdRP) in *S. shirakii*, which is consistent with a previous report that found that RdRP is not essential for simplifying dsRNA in insects [12,40]. The *enhanced RNAi-1* (*Eri-1*) gene is present in *S. shirakii*. The *Eri-1* gene is an evolutionarily-conserved gene involved in intracellular siRNA degradation.

SSRs or microsatellites are the most widely-used molecular markers, and the transcriptome sequencing is an effective method for SSR discovery [15]. These potential SSR markers identified in this paper will be valuable for genetic, evolutionary and molecular ecological applications for *S. shirakii*.

## 4. Materials and Methods

### 4.1. Species Collection, RNA Extraction and RNA-Seq

The female larval and female adult specimens of *S. shirakii* used for this study were collected in Xi’an, China. The guts of female larvae and adults were removed and then immediately frozen in liquid nitrogen, and total RNA was extracted using TRIzol reagent (Invitrogen, Carlsbad, CA, USA). After, RNA integrity was checked by the Agilent 2100 Bioanalyzer (Agilent Technologies, Mississauga, ON, Canada). Oligo (dT) magnetic beads were used to purify poly(A)^+^ RNA. Then poly(A)^+^ RNA was fragmented into small pieces at 94 °C for 5 min. First-strand cDNA synthesis used random hexamer-primer and reverse transcriptase (Invitrogen), followed by the second-strand cDNA synthesis. These cDNA fragments were treated for an end-repairing process and the ligation of adapters. Then, the products were amplified by PCR and purified by agarose gel electrophoresis and to construct a cDNA library. The mean sequence lengths of the cDNA library were 350 bp. Finally, the cDNA libraries were sequenced with pair-end 90 on the Illumina HiSeq™ 2000 platform (Illumina, San Diego, CA, USA). Raw sequence reads have been submitted to the NCBI Sequence Read Archive (Bioproject Raw sequence reads have been submitted to the NCBI Sequence Read Archive (Bioproject PRJNA295932)).

### 4.2. De Novo Assembly and Functional Annotation

Prior to assembly, low quality reads and adaptor reads were removed from raw data by using filter_fq (BGI internal software). The clean reads were then de novo assembled into unigenes by Trinity (*K* = 25) [41,42]. Next, TGICL clustering software [43] was used to process sequence clusters and resulted in unigenes.

The assembly of unigenes from *S. shirakii* was annotated by BLASTX (*E*-value < 10^−5^) against databases like Nr, Nt, Swiss-Prot, GO and KEGG with an *E*-value ≤ 10^−5^. A priority order of Nr, Swiss-Prot, KEGG and COG should be followed when results of different databases conflict with each other. The unigenes not aligned to any of the above databases were used to predict the coding regions and orientation of the sequence by the ESTScan software [16,44]. Blast2GO [16] was used to obtain GO terms of the *S. shirakii* transcriptome based on BLASTX search against the NCBI Nr database (*E*-value < 10^−5^). For the GO classifications, the default parameters were used (*E*-value < 10^−5^ and a GO weight >5).

BUSCO v.1b1 [45] was used to validate the accuracy and completeness of de novo assembly. All of the unigenes of *S. shirakii* were used to generate HMMER3 [46] hidden Markov model (HMM) profiles from amino acid alignments. Protein profiles of the *S. shirakii* unigenes for Augustus were generated with msa2prf l.pl in Augustus 3.1 [47] based on the multiple alignments. The consensus sequence of each unigene was inferred by hmmemit in the HMMER 3. The cutoff values of sequence lengths and HMMER bit scores were set according to the instruction of BUSCO v.1b1.

### 4.3. Differentially-Expressed Unigenes (DEUs)

The expressions of unigenes were normalized by using the fragments per kilobase per million (FPKM) method [48]. The differential expression levels were analyzed by the method of Audic and Claverie [49]. FDR (false discovery rate) ≤ 0.001 and |log2 Ratio| ≥ 1 were the thresholds employed to identify the significance of differential gene expression. GO functional enrichment analysis of DEUs in *S. shirakii* can acquire the results of GO functional classification annotation and enrichment analysis of DEUs. Corrected *p*-value ≤0.05 is the threshold to identify the enrichment GO term of DEUs.

### 4.4. Core RNAi Components Identification

Core RNAi homologous sequences from *L. migratoria*, *S. gregaria* and *T. castaneum* corresponding to these genes were used as a query to search the unigenes from *S. shirakii* for RNAi-related genes using the TBLASTN (ncbi-blast-2.2.24+-win32.exe) with *E*-value of 10^−5^. The RNAi components *L. migratoria*, *S. gregaria* and *T. castaneum* were as follows: AGO85968, AGO85968, AGO85968, XP_966668, XP_967454, XP_967454, XP_967454, XP_971282, XP_974696, AFY13245, AFY13245, AFY13246, AGO85972, AGO85972, AFY13246, AFQ00936, AFQ00936, AFQ00936, AFQ00936, AFQ00936, EFA02921, AGO85971, XP_001811159, EEZ99465 and JX516790. The core RNAi-related genes in *L. migratoria* or *S. gregaria* were first used as a query to search unigenes from *S. shirakii*. If the RNAi-related genes were not found with these two species, then *T. castaneum* RNAi sequences were used as the search query.

### 4.5. Identification of Genes Related to Insecticide Resistance and Phylogenetic Analysis

We identified the insecticide resistance-related sequences (P450s, CarEs and GSTs) using the BLASTX program in the Nr database with a cut-off *E*-value of 10^−5^. Contigs from the same cluster represent the same gene. If a cluster has several contigs, we just chose the longest one. Moreover, we removed the short sequences. The length of the sequences identified as putative CarEs and p450 genes <500 bp was considered to be short sequences, and the length of the sequences in GSTs <200 bp is considered to be short sequences. Some of these unigenes responded to the same gene. We identified different isoforms and transcripts based on mapping in the Nr database. The unigenes that were annotated in the same blast results were eliminated as allelic variants or as different parts of the same gene. In this case, we selected the longer unigenes.

We chose unigenes identified as P450s and *D. melanogaster* P450s superfamily protein sequences for phylogenetic analysis. Datasets for CarEs and GSTs were compiled using publicly available sequences from *L. moratoria*. Amino acid sequences were aligned using the ClustalX 2.0.10 version. The maximum likelihood trees were constructed using MEGA (Version 6.0.5, Koichiro Tamura, Tokyo, Japan) [50], employing the LG + G, WAG + I + G and LG + G amino-acid-substitution models for data of P450s, CarEs and GSTs, respectively, and node support was assessed using 1000 bootstrap replicates. The resulting trees were displayed using FigTree (Version 1.4.0, Andrew Rambaut, Edinburgh, UK) or MEGA (Version 6.0.5, Tamura K, Tokyo, Japan).

### 4.6. qRT-PCR

To validate transcriptome sequencing reliability, the same RNA samples for sequencing from adult and larval samples were used for qRT-PCR. We randomly selected seven unigenes to confirm their expressions. RNA levels were measured with the SYBR Premix ExTag quantitative PCR kit (Takara, Dalian, China), and the PCR conditions were as follows: 95 °C for 30 s, 40 cycles at 94 °C for 10 s, 60 °C for 34 s. DEUs’ expression was analyzed using CFX96 (Bio-Rad, Hercules, CA, USA). *gapdh* was used as the reference gene. The expression of the target genes relative to *gapdh* was assessed using the 2^−ΔΔ*C*t^ method. The experiments were repeated three times for each group, and the mean values were calculated. All of the primer sequences used for qRT-PCR are listed in Table S10.

### 4.7. SSRs Analysis

The MIcroSAtellite (MISA, http://pgrc.ipk-gatersleben.de/misa/) tool was used to identify SSRs with the parameters as follows: a minimum of six repeats for dinucleotide motifs, of five for trinucleotides, of four for tetranucleotides and of three for penta- and hexa-nucleotides. The SSRs with both ends on the unigene at a length of more than 150 bp were kept. The software Primer 3-2.3.4 was used to design primers.

## Figures and Tables

**Table 1:** Summary for the *S. shirakii* transcriptome after the Illumina sequencing.

Parameters	Adult	Larvae	All
Raw reads	58,063,258	62,093,046	120,156,304
Total clean reads	51,299,516	54,109,362	105,408,878
Total Nucleotides (bp)	4,616,956,440	4,869,842,580	-
Q20 ^1^ percentage (%)	97.45%	97.42%	-
N percentage (%)	0	0	-
Total length of contigs (nt)	39,306,387	54,595,446	-
Total length of unigenes (nt)	35,186,305	49,464,707	-
Number of unigenes	69,189	93,948	74,657
Mean size of contigs (nt)	290	292	-
Mean size of unigenes (nt)	509	527	680
N50 ^2^ of contigs (nt)	428	445	
N50 ^2^ of unigenes (nt)	722	800	1057
GC content (%)	47.93%	45.31%	-

^1^ The percentage of sequences at a sequencing error rate of less than 1%; ^2^ N50 is used to measure the continuity of the assembly; the greater the value, the better the assembly is. The calculation method is as follows: first, we ranked the transcripts according to their length in descending order, then accumulated one by one to 50% of the total length of all transcripts; the length of the last accumulated transcript is N50.

**Table 2:** The number of unigenes that were annotated with the databases of the NCBI non-redundant protein sequences (Nr), NCBI non-redundant nucleotide sequences (Nt), a manually annotated and reviewed protein sequence database (Swiss-Prot), Kyoto Encyclopedia of Genes and Genomes (KEGG), Clusters of orthologous group (COG) and Gene Ontology (GO).

Database Name	Nr	Nt	Swiss-Prot	KEGG	COG	GO	All
Numbers	25,652	12,671	20,438	18,053	9558	12,061	28,173

**Table 3:** Kyoto Encyclopedia of Genes and Genomes (KEGG) pathway mapping for *S. shirakii.*

KEGG Pathways	Sub-Pathways	Numbers of Unigenes
**Metabolism**	Metabolic pathways	2520
	Carbohydrate metabolism	978
	Energy metabolism	204
	Lipid metabolism	745
	Nucleotide metabolism	575
	Amino acid metabolism	895
	Metabolism of other amino acids	288
	Glycan biosynthesis and metabolism	493
	Metabolism of cofactors and vitamins	422
	Metabolism of terpenoids and polyketides	184
	Biosynthesis of other secondary metabolites	22
	Xenobiotics biodegradation and metabolism	345
**Genetic Information Processing**	Transcription	945
	Translation	1398
	Folding, sorting and degradation	1115
	Replication and repair	337
**Environmental Information Processing**	Membrane transport	313
	Signal transduction	1603
	Signaling molecules and interaction	884
**Cellular Processes**	Transport and catabolism	1262
	Cell motility	804
	Cell growth and death	597
	Cellular community	1218
**Organismal Systems**	Immune system	1307
	Endocrine system	907
	Circulatory system	475
	Digestive system	1399
	Excretory system	454
	Nervous system	806
	Sensory system	165
	Development	538
	Environmental adaptation	89

**Table 4:** Overview of identified unigenes related to the RNAi pathways in *S. shirakii*, and the RNAi-related unigene sequences are listed in the S4 Data.

Gene	Unigene	Length (bp)	*E*-Value	Identity	Species Homologue	ACCESSION
mirRNA						
*Ago-1*	Unigene6375_SsASsL	1122	0	100%	*L. migratoria*	AGO85968
	Unigene24483_SsASsL	612	7.00 × 10^−122^	100%	*L .migratoria*	AGO85968
	Unigene10467_SsASsL	444	3.00 × 10^−^^8^^2^	100%	*L. migratoria*	AGO85968
*Loqs*	Unigene5566_SsASsL	345	3.00 × 10^−12^^1^	65%	*T. castaneum*	XP_966668
*Drosha*	Unigene22829_SsASsL	339	2.00 × 10^−^^47^	78%	*T. castaneum*	XP_967454
	Unigene718_SsASsL	300	3.00 × 10^−^^67^	74%	*T. castaneum*	XP_967454
	Unigene18278_SsASsL	312	3.00 × 10^−^^5^^1^	78%	*T. castaneum*	XP_967454
*Pasha*	Unigene1479_SsASsL	672	5.00 × 10^−^^27^	59%	*T. castaneum*	XP_971282
*Exportin-5*	Unigene24927_SsASsL	1098	2.00 × 10^−1^^05^	60%	*T. castaneum*	XP_974696
siRNA						
*Dicer-2*	Unigene11038_SsASsL	531	4.00 × 10^−^^88^	87%	*S. gregaria*	AFY13245
	Unigene24754_SsASsL	237	7.00 × 10^−^^65^	78%	*S. gregaria*	AFY13245
*Ago-2*	Unigene19329_SsASsL	1737	0	88%	*S. gregaria*	AFY13246
			0	72%	*L. migratoria*	AGO85972
	Unigene24512_SsASsL	2742	0	78%	*L. migratoria*	AGO85972
			0	68%	*S. gregaria*	AFY13246
*Sid-1*	Unigene8511_SsASsL	234	4.00 × 10^−1^^4^^2^	96%	*L. migratoria*	AFQ00936
	Unigene18465_SsASsL	624	4.00 × 10^−^^99^	92%	*L. migratoria*	AFQ00936
	Unigene24347_SsASsL	474	2.00 × 10^−^^75^	99%	*L. migratoria*	AFQ00936
	Unigene10814_SsASsL	351	8.00 × 10^−^^47^	94%	*L. migratoria*	AFQ00936
	Unigene39691_SsASsL	201	7.00 × 10^−2^^9^	92%	*L. migratoria*	AFQ00936
piRNA						
*Ago-3*	Unigene11235_SsASsL	1062	4.00 × 10^−^^9^^2^	45%	*T. castaneum*	EFA02921
*Piwi3*	Unigene24609_SsASsL	2439	0	49%	*L. migratoria*	AGO85971
*Aub*	Unigene11344_SsASsL	1212	8.00 × 10^−1^^35^	54%	*T. castaneum*	XP_001811159
*Zucchini*	Unigene5197_SsASsL	684	1.00 × 10^−20^	32%	*T. castaneum*	EEZ99465
*Eri-1*	Unigene17265_SsASsL	609	4.00 × 10^−12^^1^	93%	*S. gregaria*	JX516790

## Supplementary Materials

Supplementary materials can be found at http://www.mdpi.com/1422-0067/17/7/1110/s1.

## References

[1] Cabrero J., Bugrov A., Warchalowska-Sliwa E., Lopez-Leon M.D., Perfectti F., Camacho J.P. (2003). Comparative FISH analysis in five species of Eyprepocnemidine grasshoppers. Heredity.

[2] Li C.X., Ma E.B., Zheng X.Y. (2003). Genetic differentiation of different populations of four locust species in China. Yi Chuan Xue Bao.

[3] Raymond-Delpech V., Matsuda K., Sattelle B.M., Rauh J.J., Sattelle D.B. (2005). Ion channels: Molecular targets of neuroactive insecticides. Invertebr. Neurosci..

[4] Zhang J., Yang M., Jia Q., Guo Y., Ma E., Zhu K.Y. (2011). Genomics-based approaches to screening carboxylesterase-like genes potentially involved in malathion resistance in oriental migratory locust (*Locusta migratoria manilensis*). Pest Manag. Sci..

[5] Zhang X., Wang J., Zhang M., Qin G., Li D., Zhu K.Y., Ma E., Zhang J. (2014). Molecular cloning, characterization and positively selected sites of the glutathione *S*-transferase family from *Locusta migratoria*. PLoS ONE.

[6] Ketting R.F. (2011). The many faces of RNAi. Dev. Cell.

[7] Zhang H., Li H.C., Miao X.X. (2013). Feasibility, limitation and possible solutions of RNAi-based technology for insect pest control. Insect Sci..

[8] Huvenne H., Smagghe G. (2010). Mechanisms of dsRNA uptake in insects and potential of RNAi for pest control: A review. J. Insect Physiol..

[9] Luo Y., Wang X., Yu D., Kang L. (2012). The SID-1 double-stranded RNA transporter is not required for systemic RNAi in the migratory locust. RNA Biol..

[10] Wynant N., Verlinden H., Breugelmans B., Simonet G., Vanden Broeck J. (2012). Tissue-dependence and sensitivity of the systemic RNA interference response in the desert locust, *Schistocerca gregaria*. Insect Biochem. Mol. Biol..

[11] Wynant N., Santos D., Verdonck R., Spit J., van Wielendaele P., Vanden Broeck J. (2014). Identification, functional characterization and phylogenetic analysis of double stranded RNA degrading enzymes present in the gut of the desert locust, *Schistocerca gregaria*. Insect Biochem. Mol. Biol..

[12] Tomoyasu Y., Miller S.C., Tomita S., Schoppmeier M., Grossmann D., Bucher G. (2008). Exploring systemic RNA interference in insects: A genome-wide survey for RNAi genes in Tribolium. Genome Biol..

[13] Zhang X., Allan A.C., Li C., Wang Y., Yao Q. (2015). De Novo Assembly and Characterization of the Transcriptome of the Chinese Medicinal Herb, *Gentiana rigescens*. Int. J. Mol. Sci..

[14] Zhang Y., Hao Y., Si F., Ren S., Hu G., Shen L., Chen B. (2014). The De Novo Transcriptome and Its Analysis of the Worldwide Vegetable Pest, *Delia antiqua* (Diptera: Anthomyiidae). G3 (Bethesda).

[15] Fernandez-Silva I., Whitney J., Wainwright B., Andrews K.R., Ylitalo-Ward H., Bowen B.W., Toonen R.J., Goetze E., Karl S.A. (2013). Microsatellites for next-generation ecologists: A post-sequencing bioinformatics pipeline. PLoS ONE.

[16] Conesa A., Gotz S., Garcia-Gomez J.M., Terol J., Talon M., Robles M. (2005). Blast2GO: A universal tool for annotation, visualization and analysis in functional genomics research. Bioinformatics.

[17] Tijet N., Helvig C., Feyereisen R. (2001). The cytochrome *P450* gene superfamily in *Drosophila melanogaster*: Annotation, intron-exon organization and phylogeny. Gene.

[18] Wynant N., Santos D., Vanden Broeck J. (2014). Biological mechanisms determining the success of RNA interference in insects. Int. Rev. Cell Mol. Biol..

[19] Lv J., Liu P., Gao B., Wang Y., Wang Z., Chen P., Li J. (2014). Transcriptome analysis of the *Portunus trituberculatus*: *De novo* assembly, growth-related gene identification and marker discovery. PLoS ONE.

[20] Mittapalli O., Bai X., Mamidala P., Rajarapu S.P., Bonello P., Herms D.A. (2010). Tissue-specific transcriptomics of the exotic invasive insect pest emerald ash borer (*Agrilus planipennis*). PLoS ONE.

[21] Wei D.D., Chen E.H., Ding T.B., Chen S.C., Dou W., Wang J.J. (2013). De novo assembly, gene annotation, and marker discovery in stored-product pest *Liposcelis entomophila* (Enderlein) using transcriptome sequences. PLoS ONE.

[22] Upadhyay S.K., Singh H., Dixit S., Mendu V., Verma P.C. (2016). Molecular Characterization of Vitellogenin and Vitellogenin Receptor of *Bemisia tabaci*. PLoS ONE.

[23] Wang X.W., Luan J.B., Li J.M., Bao Y.Y., Zhang C.X., Liu S.S. (2010). De novo characterization of a whitefly transcriptome and analysis of its gene expression during development. BMC Genom..

[24] Feyereisen R. (2006). Evolution of insect P450. Biochem. Soc. Trans..

[25] Claudianos C., Ranson H., Johnson R.M., Biswas S., Schuler M.A., Berenbaum M.R., Feyereisen R., Oakeshott J.G. (2006). A deficit of detoxification enzymes: Pesticide sensitivity and environmental response in the honeybee. Insect Mol. Biol..

[26] Zhang J., Li D., Ge P., Guo Y., Zhu K.Y., Ma E., Zhang J. (2014). Molecular and functional characterization of cDNAs putatively encoding carboxylesterases from the migratory locust, *Locusta migratoria*. PLoS ONE.

[27] Dabour N., Bando T., Nakamura T., Miyawaki K., Mito T., Ohuchi H., Noji S. (2011). Cricket body size is altered by systemic RNAi against insulin signaling components and epidermal growth factor receptor. Dev. Growth Differ..

[28] Belles X. (2010). Beyond *Drosophila*: RNAi in vivo and functional genomics in insects. Annu. Rev. Entomol..

[29] Karatolos N., Williamson M.S., Denholm I., Gorman K., Ffrench-Constant R.H., Bass C. (2012). Over-expression of a cytochrome P450 is associated with resistance to pyriproxyfen in the greenhouse whitefly *Trialeurodes vaporariorum*. PLoS ONE.

[30] Edi C.V., Djogbenou L., Jenkins A.M., Regna K., Muskavitch M.A., Poupardin R., Jones C.M., Essandoh J., Ketoh G.K., Paine M.J. (2014). CYP6 P450 enzymes and ACE-1 duplication produce extreme and multiple insecticide resistance in the malaria mosquito *Anopheles gambiae*. PLoS Genet..

[31] Guo Y., Wu H., Zhang X., Ma E., Guo Y., Zhu K.Y., Zhang J. (2016). RNA interference of cytochrome P450 CYP6F subfamily genes affects susceptibility to different insecticides in *Locusta migratoria*. Pest Manag. Sci..

[32] Grubor V.D., Heckel D.G. (2007). Evaluation of the role of CYP6B cytochrome P450s in pyrethroid resistant Australian *Helicoverpa armigera*. Insect Mol. Biol..

[33] Zhang X., Liu X., Ma J., Zhao J. (2013). Silencing of cytochrome P450 *CYP6B6* gene of cotton bollworm (*Helicoverpa armigera*) by RNAi. Bull. Entomol. Res..

[34] Tang T., Zhao C., Feng X., Liu X., Qiu L. (2012). Knockdown of several components of cytochrome P450 enzyme systems by RNA interference enhances the susceptibility of *Helicoverpa armigera* to fenvalerate. Pest Manag. Sci..

[35] Zhang J., Li D., Ge P., Yang M., Guo Y., Zhu K.Y., Ma E. (2013). RNA interference revealed the roles of two carboxylesterase genes in insecticide detoxification in *Locusta migratoria*. Chemosphere.

[36] Xu L., Duan X., Lv Y., Zhang X., Nie Z., Xie C., Ni Z., Liang R. (2014). Silencing of an aphid carboxylesterase gene by use of plant-mediated RNAi impairs *Sitobion avenae* tolerance of Phoxim insecticides. Transgenic Res..

[37] Kola V.S., Renuka P., Madhav M.S., Mangrauthia S.K. (2015). Key enzymes and proteins of crop insects as candidate for RNAi based gene silencing. Front. Physiol..

[38] Winston W.M., Molodowitch C., Hunter C.P. (2002). Systemic RNAi in *C. elegans* requires the putative transmembrane protein SID-1. Science.

[39] Xu H.J., Chen T., Ma X.F., Xue J., Pan P.L., Zhang X.C., Cheng J.A., Zhang C.X. (2013). Genome-wide screening for components of small interfering RNA (siRNA) and micro-RNA (miRNA) pathways in the brown planthopper, *Nilaparvata lugens* (Hemiptera: Delphacidae). Insect Mol. Biol..

[40] Tribolium Genome Sequencing C., Richards S., Gibbs R.A., Weinstock G.M., Brown S.J., Denell R., Beeman R.W., Gibbs R., Beeman R.W., Brown S.J. (2008). The genome of the model beetle and pest Tribolium castaneum. Nature.

[41] Grabherr M.G., Haas B.J., Yassour M., Levin J.Z., Thompson D.A., Amit I., Adiconis X., Fan L., Raychowdhury R., Zeng Q. (2011). Full-length transcriptome assembly from RNA-Seq data without a reference genome. Nat. Biotechnol..

[42] Haas B. J., Papanicolaou A., Yassour M., Grabherr M., Blood P.D., Bowden J., Couger M.B., Eccles D., Li B., Lieber M. (2013). *De novo* transcript sequence reconstruction from RNA-seq using the Trinity platform for reference generation and analysis. Nat. Protoc..

[43] Pertea G., Huang X., Liang F., Antonescu V., Sultana R., Karamycheva S., Lee Y., White J., Cheung F., Parvizi B. (2003). TIGR Gene Indices clustering tools (TGICL): A software system for fast clustering of large EST datasets. Bioinformatics.

[44] Iseli C., Jongeneel C.V., Bucher P. (1999). ESTScan: A program for detecting, evaluating, and reconstructing potential coding regions in EST sequences. ISMB.

[45] Simao F.A., Waterhouse R.M., Ioannidis P., Kriventseva E.V., Zdobnov E.M. (2015). BUSCO: Assessing genome assembly and annotation completeness with single-copy orthologs. Bioinformatics.

[46] Eddy S.R. (2011). Accelerated Profile HMM Searches. PLoS Comput. Biol..

[47] Keller O., Kollmar M., Stanke M., Waack S. (2011). A novel hybrid gene prediction method employing protein multiple sequence alignments. Bioinformatics.

[48] Mortazavi A., Williams B.A., McCue K., Schaeffer L., Wold B. (2008). Mapping and quantifying mammalian transcriptomes by RNA-Seq. Nat. Methods.

[49] Audic S., Claverie J.M. (1997). The significance of digital gene expression profiles. Genome Res..

[50] Tamura K., Stecher G., Peterson D., Filipski A., Kumar S. (2013). MEGA6: Molecular Evolutionary Genetics Analysis version 6.0. Mol. Biol. Evol..

