# Supplementary material for: De Novo Assembly and Characterization of the Transcriptome of Grasshopper Shirakiacris shirakii
Source: Int J Mol Sci. 2016 Jul 22;17(7):1110. doi: 10.3390/ijms17071110 (PMC4964485; doi:10.3390/ijms17071110)
Supplement: Supplementary file 1 [file ijms-17-01110-s001.zip › ijms-127851-Supplementary Materials/ijms-127851-supplementary figures-final.pdf]

# Supplementary Materials: De Novo Assembly and Characterization of the Transcriptome of Grasshopper *Shirakiacris shirakii*

**Zhongying Qiu, Fei Liu, Huimeng Lu, Hao Yuan, Qin Zhang and Yuan Huang**

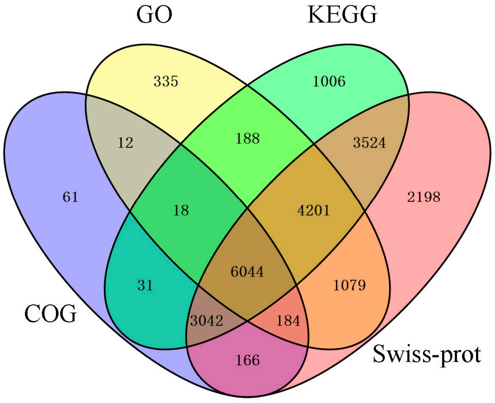


**Figure S1.** The Venn diagram of the COG, GO KEGG and Swiss-prot databases.

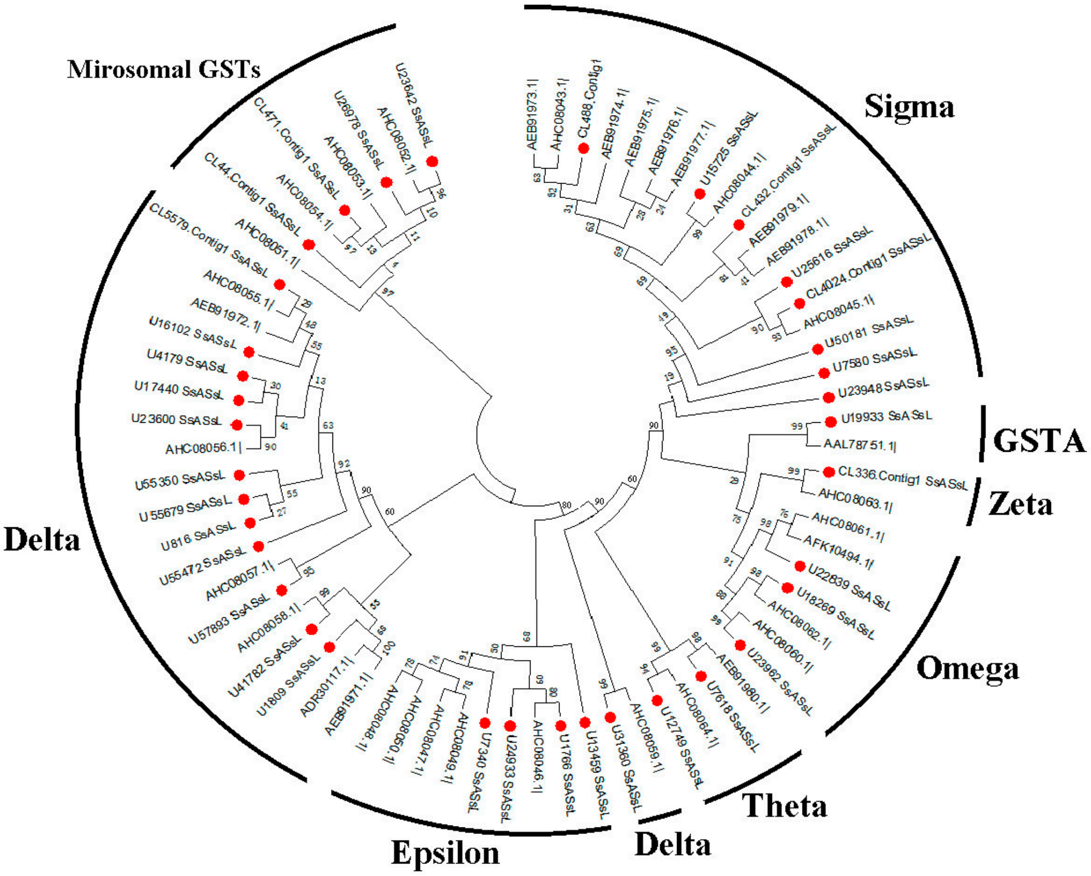


**Figure S2.** The phylogenetic analysis of GSTs-encoding genes from *S. shirakii* and *L. migtatoria*. Branch numbers represent bootstrap values (1000 replicates). The 36 unigenes encoding GSTs were marked with red filled circles. The sequences used to reconstruct the maximum likelihood (ML) tree were available as S3 Data.
